# Long-acting beta-agonists reduce mortality of patients with severe and very severe chronic obstructive pulmonary disease: a propensity score matching study

**DOI:** 10.1186/1465-9921-14-62

**Published:** 2013-06-03

**Authors:** Nobuyuki Horita, Naoki Miyazawa, Satoshi Morita, Ryota Kojima, Naoko Kimura, Takeshi Kaneko, Yoshiaki Ishigatsubo

**Affiliations:** 1 Department of Internal Medicine and Clinical Immunology, Yokohama City University Graduate School of Medicine, Yokohama, Japan; 2 Department of Respiratory Medicine, Saiseikai Yokohamashi Nanbu Hospital, Yokohama, Japan; 3 Department of Biostatistics and Epidemiology, Yokohama City University Medical Center, Yokohama, Japan; 4 Respiratory Disease Center, Yokohama City University Medical Center, Yokohama, Japan

**Keywords:** Bronchodilator agents, Treatment outcome, Case-control study, Propensity score

## Abstract

**Background:**

Long-acting beta-agonists were one of the first-choice bronchodilator agents for stable chronic obstructive pulmonary disease. But the impact of long-acting beta-agonists on mortality was not well investigated.

**Methods:**

National Emphysema Treatment Trial provided the data. Severe and very severe stable chronic obstructive pulmonary disease patients who were eligible for volume reduction surgery were recruited at 17 clinical centers in United States during 1988–2002. We used the 6–10 year follow-up data of patients randomized to non-surgery treatment. Hazard ratios for death by long-acting beta-agonists were estimated by three models using Cox proportional hazard analysis and propensity score matching were measured.

**Results:**

The pre-matching cohort was comprised of 591 patients (50.6% were administered long-acting beta-agonists. Age: 66.6 ± 5.3 year old. Female: 35.4%. Forced expiratory volume in one second (%predicted): 26.7 ± 7.1%. Mortality during follow-up: 70.2%). Hazard ratio using a multivariate Cox model in the pre-matching cohort was 0.77 (P = 0.010). Propensity score matching was conducted (C-statics: 0.62. No parameter differed between cohorts). The propensity-matched cohort was comprised of 492 patients (50.0% were administered long-acting beta-agonists. Age: 66.8 ± 5.1 year old. Female: 34.8%. Forced expiratory volume in one second (%predicted) 26.5 ± 6.8%. Mortality during follow-up: 69.1%). Hazard ratio using a univariate Cox model in the propensity-matched cohort was 0.77 (P = 0.017). Hazard ratio using a multivariate Cox model in the propensity-matched cohort was 0.76 (P = 0.011).

**Conclusions:**

Long-acting beta-agonists reduce mortality of severe and very severe chronic obstructive pulmonary disease patients.

## Background

Long-acting beta-agonists (LABA), one of the first-choice bronchodilator agents for stable chronic obstructive pulmonary disease (COPD) [1] improve obstruction, hyperinflation, quality of life, dyspnea, frequency of exacerbation, and frequency of rescue medication [2-6]. Changes in the quality of life, pulmonary function, and other parameters were often used as surrogate endpoints to evaluate the effect of agents, because it is often difficult to observe sufficient deaths in feasible studies. Considering favorable outcomes about surrogate endpoints, it is reasonable to hypothesize that LABA improve life prognosis of stable COPD patients. However these surrogate endpoints do not always reflect mortality of respiratory disease with airflow obstruction. One famous example is beta-agonist for bronchial asthma. The beta-agonists improve airflow obstruction, dyspnea, and quality of life of bronchial asthma patients. However, the regular use of inhaled beta-agonist increasing the number of deaths or near-death condition from bronchial asthma [7]. Thus, whether LABA actually reduce the mortality of COPD patients is an important question despite the repeatedly proven association between lung function, quality of life, and LABA administration for COPD patients.

In 2007, Calverley et al conducted a large-scale randomized controlled trial spanning 3 years, involving 6112 patients, in which mortality as secondary endpoint was compared between placebo- and LABA-administrated cohorts [3]. However, their study showed no significant reduction in mortality, and the authors concluded that the study was not an accurate reflection of the mortality because of high withdrawal rate and fewer observed deaths than anticipated.

Therefore, in this study, we evaluated the life prognosis of patients treated with LABA in a cohort with prospectively collected data using Cox hazard model and propensity score matching method.

## Methods

The data set previously collected for the National Emphysema Treatment Trial (NETT) [8] was provided by the National Heart, Lung, and Blood Institute. The current study was approved by the Yokohama City Hospital Institutional Review Board. The need for informed consent was waived for this study due to patient anonymity and the observational nature of the study.

**Figure 1:**
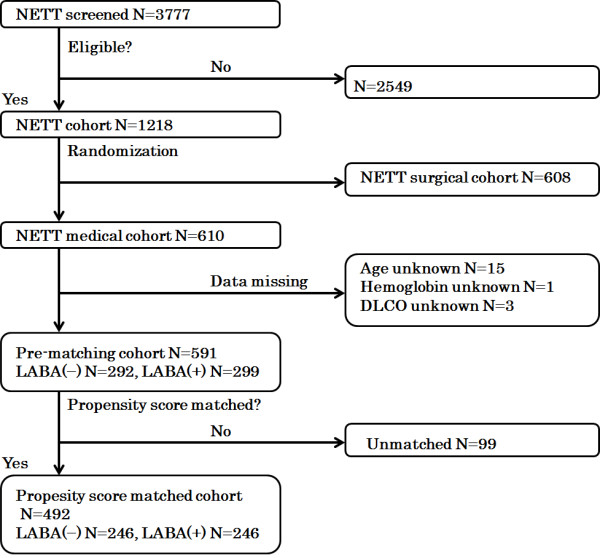
**Flow chart for patient entry.** LABA: Long-acting beta-agonist. NETT: National emphysema treatment trial.

We calculated the hazard ratios (HRs) for death from LABA administration in three Cox proportional hazards models. In Model 1, we evaluated the HR using a multivariate Cox model in the pre-matching cohort. Propensity score matching was performed before the analyses for Models 2 and 3. In Model 2, we estimated the HR using a univariate Cox model in a propensity-matched cohort. In Model 3, we evaluated the HR using a multivariate Cox model in the propensity-matched cohort. Our primary outcome was death, evaluated as HR in the three models (Figure 1, Table 1).

**Table 1:** Comparison of three independent models

**Model**	**1**	**2**	**3**
Propensity score matched?	No	Yes	Yes
Any difference between cohorts?	Yes (not much)	No	No
Single- or multi-variable analysis?	Multi-	Single-	Multi-
Number of patients	591	492	492

### Patient selection

The major entry criteria for the NETT study were as follows: radiographic evidence of bilateral emphysema; forced expiratory volume in one second (FEV1) (%predicted) ≤ 45%; a pressure of oxygen in artery (PaO_2_) ≥ 45 mmHg; a pressure of carbon dioxide in artery (PaCO_2_) ≤ 60 mmHg; 6-minute walking distance ≥ 140 m; participation in pulmonary rehabilitation; not at high risk for perioperative morbidity or mortality; suitable for lung volume reduction surgery; and likeliness to completing the trial. From January 1998 to July 2002, 3777 patients were evaluated in 17 clinical centers; of the 1218 patients eligible for enrollment, 610 and 608 were randomly allocated to the medical and surgical cohorts respectively. The inclusion criteria are described in more detail in the previous report [8]. We used the data of only medical cohort patients in this study. Among the 610 patients, patients who lacked data for age (N = 15), hemoglobin (N = 1), or diffusing capacity of the lung for carbon monoxide (DLCO) (N = 3) were excluded. The remaining 591 patients comprised the *pre-matching cohort* and the propensity-matched 492 patients comprised the *propensity-matched cohort* (Figure 1). A patient who was or was not prescribed LABA at randomization (= 0 months) was considered a LABA(+) or LABA(−) patient.

### Treatments

The primary care physicians provided the following treatment in close compliance with the guideline [9]: smoking cessation, regular inhaled bronchodilators, oxygen therapy, immunization, pulmonary rehabilitation, and additional measures including oral corticosteroids [8].

### Measurements

Spirometric data were collected after administering bronchodilators. The DLCO was adjusted for hemoglobin: DLCO × hemoglobin/0.0697. PaO_2_ and PaCO_2_ were measured in ambient air even if patients were administered long-term oxygen therapy (LTOT). The cutoff value for an emphysematous change on CT was −950 Hounsfield. The peak pulmonary arterial pressure was measured using right heart catheterization or an echocardiography. The peak pulmonary arterial pressure measured by echocardiography was estimated as follows: mean right atrial pressure + 4 × (estimated tricuspid peak systolic velocity)^2. Recent emergency hospital stay included both admission and overstay in the hospital. “Recent” emergency visit and “recent” hospital stay indicated events during the last three months were asked on pre-observational data acquisition. Death was defined as death due to all causes, not just respiratory-related deaths. Additional information about the measurement method is provided previously [8].

### LABA prescription during the follow-up

We checked whether the patients were administered LABA or not at 6, 12, 24, 46, 48 and 60 months. This was evaluated only for interpretation of endpoints (i.e., the intention-to-treat principle).

### Statistics

The Chi-square test (with Yates’ correction, if necessary), the Wilcoxon rank sum test, the Cox proportional hazard analysis, Kaplan-Meier analysis, and the logrank test were used where appropriate. Propensity score matching is a matching technique to balance several variables at once by predicting the probability that each patient receive some intervention or not. The most important advantage of propensity score matching over classical matching is that this method can balance much more variables efficiently, whereas the classical matching usually cannot balance no more than 10 parameters due to deficit of cases. Detailed methodology for this matching is provided in other article [10]. We used three different models and believe that the consistency among the models enhances the validity of study results [11,12] (Table 1). Propensity score matching was performed to balance 26 independent variables. The pre-matching cohort was divided into five subgroups according to the propensity score and the maximum number of matches possible was made in each subgroup with computer-based randomization [10]. Each LABA(+) patient was matched with one LABA (−) patient. The quality of matching was evaluated by comparing patient characteristics between cohorts. The area of emphysematous change (%) and peak pulmonary arterial pressure were used only for comparison between LABA (+) and LABA (−) cohorts and not for multivariate analysis as data for some patients were not available. Forced vital capacity (FVC) (mL), FEV1 (mL), and FEV1/FVC (%) were also excluded from multivariate analysis to avoid possible multicolinearity with FVC (%predicted) and/or FEV1 (%predicted).

## Results and discussion

### Pre-matching cohort

**Figure 2:**
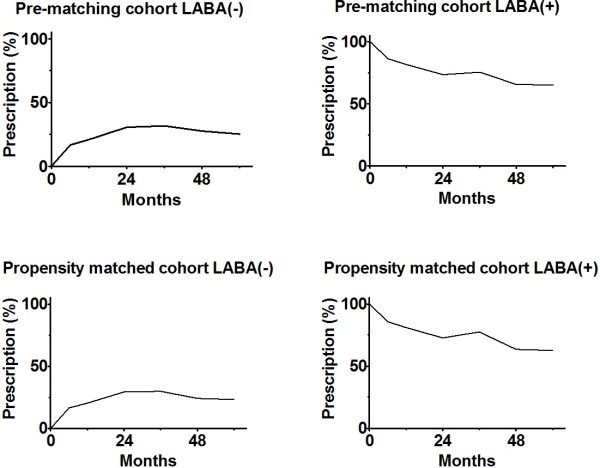
**Prescription of LABA (long-acting beta-agonist) during follow-up. **On 0 month, there were 292 LABA (−) patients and 299 LABA (+) patients in the pre-matching cohort; and 246 LABA (−) patients and 246 LABA (+) patients in the propensity matched cohort.

The background characteristics of the 591 patients in the pre-matching cohort are summarized in Table 2. In general, cohorts of 292 LABA (−) patients and 299 LABA (+) patients had similar characteristics except for short-acting beta-agonist administration. In the pre-matching cohort, 176 patients survived for the observation period, and the duration until censoring was 94.7 ± 14.4 (range 70.1–123.9) months. Throughout the observation period, ≥ 65% of LABA (+) and (−) patients were treated with the initially classified treatments (Figure 2).

**Table 2:** Baseline characteristics of patients (pre-matching cohort)

	**All patients**	**Comparison of LABA (+) and (−) cohorts**		
		**LABA (−)**	**LABA (+)**	**P**
N	591	292	299	
Age (year)	66.6 ± 5.3	66.9 ± 5.2	66.4 ± 5.3	0.208
Sex (female)	209 (35.4%)	93 (31.8%)	116 (38.8%)	0.074
Race (not white)	33 (5.6%)	20 (6.8%)	13.0 (4.3%)	0.185
Annual income < 30000 $	304 (51.4%)	155 (53.1%)	149 (49.8%)	0.429
FEV1 (%predicted)	26.7 ± 7.1	26.8 ± 7.1	26.7 ± 7.0	0.780
FEV1 (mL)	777 ± 240	782 ± 237	772 ± 243	0.456
FEV1/FVC (%)	31.2 ± 6.3	30.9 ± 6.0	31.5 ± 6.6	0.358
FVC (%predicted)	67.2 ± 15.1	68.0 ± 15.3	66.5 ± 14.9	0.346
FVC (mL)	2545 ± 782	2582 ± 772	2509 ± 791	0.225
Forced residual capacity (mL)	6042 ± 1311	6111 ± 1323	5975 ± 1298	0.243
Hb adjusted DLCO	8.1 ± 3.1	8.1 ± 3.0	8.0 ± 3.1	0.761
Peak pulmonary arterial pressure (mmHg)	33.7 ± 6.2 (N = 502)	34.2 ± 6.1 (N = 252)	33.3 ± 6.3 (N = 250)	0.110
Hemoglobin (g/dL)	14.4 ±1.3	14.4 ± 1.2	14.3 ± 1.3	0.905
PaO_2_ (mmHg)	64.2 ± 10.0	64.0 ±10.0	64.4 ± 10.0	0.838
PaCO_2_ (mmHg)	43.1 ± 5.8	43.6 ± 6.1	42.6 ± 5.4	0.083
Area of emphysema (%)	16.1 ± 10.6 (N = 532)	15.7 ± 11.1 (N = 259)	16.4 ± 10.2 (N = 273)	0.228
Body mass index (kg/m^2)	24.7 ± 3.5	24.7 ± 3.6	24.8 ± 3.5	0.503
Six-minute walk distance	368 ± 96	366 ± 95	370 ± 98	0.562
St. George respiratory questionnaire	53.6 ± 12.7	54.1 ± 12.6	53.1 ± 12.7	0.394
Shortness of breath questionnaire	63.3 ± 18.5	63.8 ± 18.1	62.7 ± 18.9	0.483
Beck depression inventory	9.3 ± 5.9	9.2 ± 5.8	9.3 ± 5.9	0.650
Recent emergency visit	75 (12.7%)	38 (13.0%)	37 (12.4%)	0.816
Recent hospital stay	48 (8.1%)	26 (8.9%)	22 (7.4%)	0.492
LTOT during sleep	414 (70.1%)	206 (70.5%)	208 (69.6%)	0.794
LTOT on exertion	465 (78.7%)	233 (79.8%)	232 (77.6%)	0.513
Oral corticoid	153 (25.9%)	76 (26.0%)	77 (25.8%)	0.939
Inhaled corticosteroid	447 (75.6%)	211 (72.3%)	236 (78.9%)	0.059
Short-acting beta-agonist	515 (87.1%)	264 (90.4%)	251 (83.9%)	0.019
Anticholinergic agent	486 (82.2%)	242 (82.9%)	244 (81.6%)	0.686
Theophylline	238 (40.3%)	124 (42.5%)	114 (38.1%)	0.282
Diuretics	86 (14.6%)	41 (14.0%)	45 (15.1%)	0.728

Parameters were compared with Wilcoxon rank sum test or Chi-square test.

LABA: long-acting beta-agonist.

FEV1: forced expiratory volume in one second.

FVC: forced vital capacity.

Hb adjusted DLCO: hemoglobin adjusted diffusing capacity of the lung for carbon monoxide.

PaO_2_: pressure of oxygen in artery.

PaCO_2_: pressure of carbon dioxide in artery.

LTOT: long-term oxygen therapy.

### Model 1

**Figure 3:**
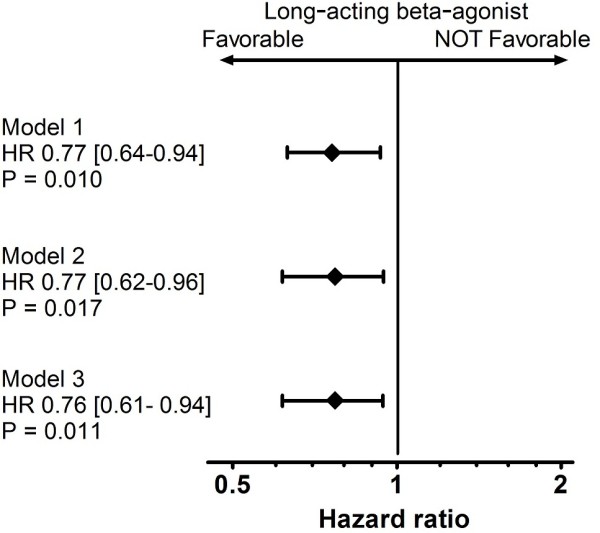
Hazard ratio by long-acting beta-agonists for death.

The stepwise multivariable Cox model analysis which initially included LABA and 26 other covariables as independent variables was performed in the pre-matching cohort. Eleven independent variables remained in the last model. The HR for death due to LABA use was 0.77 (95% CI: 0.64–0.94; P = 0.010) (Figure 3).

### Propensity score matched cohort

Logistic regression analysis was performed for propensity matching. Only non-administration of short-acting beta-agonist (P = 0.039) and decreased PaCO_2_ level (P = 0.007) were significantly associated with LABA administration in this model. The mean propensity scores among LABA (−) and LABA (+) patients were 0.48 ± 0.11 and 0.53 ± 0.11, respectively. The C-statistic was 0.62. The pre-matching cohort was divided into five subgroups on the basis of the propensity score. Among the five subgroups, 246 LABA(+) patients were matched with 246 LABA(−) patients. There was no significant difference between the cohorts (Table 3). Throughout the observation period, ≥ 63% of LABA(+) and > 70% of LABA(−) patients were treated with the initially classified treatments (Figure 2).

**Table 3:** Comparison of LABA (+) and LABA (−) patients in the propensity-matched cohort

	**LABA (−)**	**LABA (+)**	
N	246	246	P
Age (year)	66.7 ± 5.0	66.8 ± 5.3	0.732
Sex (female)	86 (35.0%)	85 (34.6%)	0.925
Race (not white)	10 (4.1%)	12 (4.9%)	0.663
Annual income < 30000 $	125 (50.8%)	123 (50.0%)	0.857
FEV1 (%predicted)	26.5 ± 6.8	26.6 ± 6.8	0.991
[FEV1 (mL)]	766 ± 222	777 ± 242	0.866
[FEV1/FVC (%)]	31.0 ± 6.0	31.2 ± 6.3	0.926
FVC (%predicted)	67.1 ± 15.4	67.0 ± 15.0	0.849
[FVC (mL)]	2527 ± 757	2556 ± 802	0.772
Forced residual capacity (mL)	6072 ± 1330	6040 ± 1306	0.842
Hb adjusted DLCO	7.9 ± 2.9	8.1 ± 3.2	0.632
[Peak pulmonary arterial pressure(mmHg)]	34.0 ± 6.0	33.3 ± 6.2	0.202
Hemoglobin (g/dL)	14.4 ± 1.2	14.4 ± 1.3	0.626
PaO_2_ (mmHg)	64.2 ± 10.0	64.2 ± 10.2	0.829
PaCO_2_ (mmHg)	43.1 ± 5.8	43.0 ± 5.4	0.972
[Area of emphysema (%)]	16.3 ± 11.1	16.0 ± 9.5	0.759
Body mass index (kg/m^2)	24.7 ± 3.5	24.7 ± 3.6	0.860
Six-minute walk distance (m)	366 ± 93	371 ± 98	0.577
St. George respiratory questionnaire	53.4 ± 12.5	53.3 ± 12.6	0.974
Shortness of breath questionnaire	63.0 ± 17.9	63.0 ± 19.0	0.961
Beck depression inventory	9.1 ± 5.8	9.0 ± 5.8	0.988
Recent emergency visit	33 (13.4%)	30 (12.2%)	0.686
Recent hospital stay	19 (7.7%)	17 (6.9%)	0.729
LTOT during sleep	173 (70.3%)	170 (69.1%)	0.769
LTOT on exertion	191 (77.6%)	191 (77.6%)	1.000
Oral corticoid	70 (28.5%)	62 (25.2%)	0.416
Inhaled corticosteroid	186 (75.6%)	186 (75.6%)	1.000
Short-acting beta-agonist	219 (89.0%)	217 (88.2%)	0.777
Anticholinergic agent	205 (83.3%)	203 (82.5%)	0.811
Theophylline	103 (41.9%)	94 (38.2%)	0.408
Diuretics	34 (13.8%)	38 (15.4%)	0.610

[parameters]: parameters not used in logistic regression analysis for propensity score matching were shown in brackets.

Parameters were compared with Wilcoxon rank sum test or Chi-square test.

FEV1: forced expiratory volume in one second.

FVC: forced vital capacity.

Hb adjusted DLCO: hemoglobin adjusted diffusing capacity of the lung for carbon monoxide.

PaO_2_: pressure of oxygen in artery.

PaCO_2_: pressure of carbon dioxide in artery.

LTOT: long-term oxygen therapy.

### Model 2

**Figure 4:**
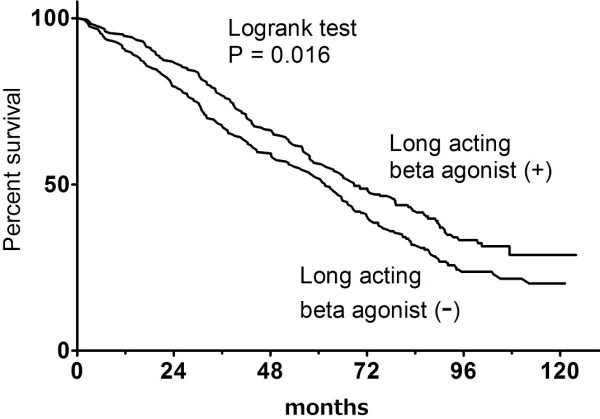
Kaplan meier curve for survival (Model 2).

In the propensity-matched cohort, univariate Cox model analysis produced HR for death from LABA of 0.77 (95%CI: 0.62–0.96; P = 0.018) (Figure 3). The Kaplan-Meier curve showed that LABA(+) patients had better life prognosis than LABA (−) patients (P = 0.016, Logrank test) (Figure 4).

### Model 3

The stepwise multivariable Cox model analysis, which initially included LABA and 26 other co-variables as independent variables, was performed in the propensity-matched cohort. Eleven independent variables remained in the last model. The HR for death by LABA was 0.76 (95%CI: 0.61–0.94; P = 0.011) (Figure 4).

### Discussion

To our knowledge, this is the first study demonstrating that LABA reduce mortality of COPD patients. Although the reason for this mortality reduction is unclear, LABA may prevent further deterioration of conditions by reducing the frequency of exacerbation, and may prevent pulmonary hypertension and cor pulmonale by improving lung functions [2-6].

Our results are not in complete agreement with those of the well-designed large-scale randomized controlled trial by Calverley [3]. A few possible reasons for this discrepancy are as follows: (i) Calverley observed 231 deaths (15.2%) among 1524 placebo patients and 205 deaths (13.5%) among1521 salmeterol patients, whereas in our pre-matching cohort, we observed 222 deaths (76.0%) among 292 LABA (−) patients and 193 deaths (64.5%) among 299 LABA (+) patients. Although our cohort is smaller than that in the Calverley’s study, the higher mortality enabled us to observe similar numbers of deaths. The higher mortality is mainly supported by the two reasons: FEV1(%predicted) was 44% ± 12% in the Calverley’s and 27% ± 7% in ours; Calverley’s included data of 3 years, whereas the mean observational duration for survivors was 7.9 years in ours.(ii) Statistical power in our study is also supported by higher “compliance” to the classified protocol. At the 36th month, less than 32% of LABA (−) patients were administered LABA and less than 25% of the LABA (+) patients were not administered LABA. Contrarily, withdrawal rate in placebo cohort in Calverley’s study was 44% after three years. Some researchers performed factorial analysis on Calverley’s study and concluded that salmeterol was associated with decreased mortality with relative risk of 0.83 (P = 0.004), which is compatible with our study [13,14].

The results of the meta-analysis of Salpeter et al in 2006 conflict with our result [15]. They reported that 21 (1.6%) of 1320 beta-agonist administered patients had respiratory-related death, which is significantly higher than mortality of 0.7% (8 of 1084) among beta-agonist non-administered patients. Only 1.2% of patients in the meta-analysis died, partly because the observation period was as short as 3 to 12 months and because respiratory-related death (not all-cause death) was adopted as an endpoint in their study. COPD patients often died from cardiovascular disease, which was perhaps exacerbated by systemic inflammation or cor pulmonale [16]. All-cause death, not just respiratory death, is desirable for evaluating deaths due to co-morbidities. Furthermore, 19 of 29 deaths in the meta-analysis were observed in one unpublished study. If Calverley’s large scale study in 2007 [3] had also been included in this meta-analysis, the results would have favored beta-agonist administration.

Our study has several limitations. First, this was an observational study and not a randomized controlled trial. Since the current guideline [1] recommends administering LABA, a randomized controlled trial was not thought to be a feasible. Our study design was a reasonable choice to evaluate the effect of LABA among severe and very severe COPD patients. Second, our study involved “post-treatment variables.” For propensity matching, covariates had better be evaluated before the dependent variable. This implies that the physician should have decided whether or not to start LABA after all parameters were measured. However, this assumption was not fully satisfied in this study. LABA administered before pre-observational data acquisition affects baseline characteristics in this study. If the patients previously received LABA treatment, it is likely that the improvement or deterioration in their condition already occurred at the time of pre-observational data acquisition. The LABA (+) and (−) cohorts in the propensity-matched cohort were equal (Table 3) at pre-observational data acquisition because of cancellation of the treatment effect of LABA before propensity score matching. This bias impaired the observed effect of LABA and increased beta error. Though, we observed significant HR. Third, the type of LABA is not clearly identified in our study, because NETT database groups all LABAs in a single category. Only salmeterol was approved in the United States on the beginning of this study. Formoterol and arformoterol were additionally approved in 2001 and 2006. Considering market share during 1998-2008, salmeterol, which is often prescribed as fluticasone/salmeterol combination, was prescribed for most of the patients; and formoterol or arformoterol were prescribed for small portion of patients. Recently used once daily indacaterol was not approved until 2011, which is after end of data collection of this study. Fourth, our cohort contained only patients with FEV1 (%predicted) ≤ 45% and this study did not clarify if LABA improves the life prognosis of COPD patients with FEV1 (%predicted) > 45. However, we still believe LABA have favorable effects on mild and moderate COPD patients, because LABA improve obstruction, hyperinflation, quality of life, dyspnea, frequency of exacerbation, and frequency of rescue medication among mild and moderate COPD patients [2-6], and because LABA reduce mortality among severe and very severe COPD patients as shown in this study. Fifth, about 30% of patients were “deviated” from the initial treatment after 3 years (Figure 2). But we do not think these deviations are not serious problem, because these treatment deviation cause bias to distract the difference of observed mortality, i.e. HR getting closer to 1.0. Nonetheless, our study find out statistically and clinically significant difference among LABA (+) and LABA (−) patients. Lastly, we cannot evaluate respiratory-related mortality, because data about cause of death was not provided.

## Conclusion

We demonstrated that LABAs, mostly salmeterol, reduce death among severe and very severe COPD patients with a HR of 0.76–0.77.

## Abbreviations

COPD: Chronic obstructive pulmonary disease; LABA: Long-acting beta-agonist; LTOT: Long-term oxygen therapy; HR: Hazard ratio; FEV1: Forced expiratory volume in one second; FVC: Forced vital capacity; DLCO: Diffusing capacity of the lung for carbon monoxide; PaO2: Pressure of oxygen in artery; PaCO2: Pressure of carbon dioxide in artery; NETT: National emphysema treatment trial.

## Competing interests

Authors have no conflict of interest.

## Authors’ contribution

All authors contributed conception, design, data acquisition, analysis, interpretation, drafting, revising, and final approval of the manuscript. Nobuyuki Horita served as a principal investigator and a guarantor and had full access to all of the data in the study and takes responsibility for the integrity of the data and the accuracy of the data analysis. Naoki Miyazawa, Ryota Kojima and Naoko Omori provided interpretation of data and drafting. Satoshi Morita worked as statistician. Takeshi Kaneko and Yoshiaki Ishigatsubo provided study management. All authors read and approved the final manuscript.
